# The Structural Basis for Promoter −35 Element Recognition by the Group IV σ Factors

**DOI:** 10.1371/journal.pbio.0040269

**Published:** 2006-08-15

**Authors:** William J Lane, Seth A Darst

**Affiliations:** The Rockefeller University, New York, New York, United States of America; University of California San Diego, United States of America

## Abstract

The control of bacterial transcription initiation depends on a primary σ factor for housekeeping functions, as well as alternative σ factors that control regulons in response to environmental stresses. The largest and most diverse subgroup of alternative σ factors, the group IV extracytoplasmic function σ factors, directs the transcription of genes that regulate a wide variety of responses, including envelope stress and pathogenesis. We determined the 2.3-Å resolution crystal structure of the −35 element recognition domain of a group IV σ factor, Escherichia coli σ^E^_4_, bound to its consensus −35 element, GGAACTT. Despite similar function and secondary structure, the primary and group IV σ factors recognize their −35 elements using distinct mechanisms. Conserved sequence elements of the σ^E^ −35 element induce a DNA geometry characteristic of AA/TT-tract DNA, including a rigid, straight double-helical axis and a narrow minor groove. For this reason, the highly conserved AA in the middle of the GGAACTT motif is essential for −35 element recognition by σ^E^_4_, despite the absence of direct protein–DNA interactions with these DNA bases. These principles of σ^E^_4_/−35 element recognition can be applied to a wide range of other group IV σ factors.

## Abstract 2

A crystal structure of *E. coli* Group IV σ factor bound to DNA identifies a distinct mechanism of interaction reliant on the rigid conformation of the DNA consensus sequence.

## Introduction

Bacterial transcription is driven by the DNA-dependent RNA polymerase (RNAP), comprising five core subunits (α_2_ββ′ω) plus an initiation-specific σ subunit, which binds to the core RNAP to form the holoenzyme [1–3]. Promoter-specific transcription initiation first requires the formation of a closed complex in which σ domains 2 (σ_2_) and 4 (σ_4_) bind sequence-specifically to the −10 and −35 promoter DNA elements, respectively [3–5]. Analysis of the available bacterial genomes has revealed great variation in both the number and type of σ factors that each bacterial species possesses [6,7], allowing for promoter-specific transcription of defined regulons.

Most σ factors belong to the σ^70^ family, which can be broadly divided into five subgroups [7,8]. The group I (primary) σ factors, such as *Escherichia coli (Ec)* σ^70^ and *Thermus aquaticus (Taq)* σ^A^, direct the transcription of housekeeping genes for which basal levels of transcription are essential for normal cellular processes and survival. The largest and most diverse subgroup, the group IV, or extracytoplasmic function (ECF) σ factors, direct the transcription of genes that regulate a wide variety of responses including periplasmic stress, iron transport, metal ion efflux, alginate secretion, and pathogenesis [7,9–11]. The *Ec* ECF σ factor σ^E^ is an essential protein that directs the response to periplasmic stress [12–15].

Like many ECF σs, *Ec* σ^E^ is regulated by an anti-σ, RseA [13,15]. Under normal conditions, RseA inactivates σ^E^ by sequestering it at the cytoplasmic face of the inner membrane. However, when environmental stresses lead to unfolded proteins in the periplasm, a series of proteolytic cleavage reactions release σ^E^ from RseA [16]. The σ^E^ is then free to bind RNAP and drive the transcription of a core set of genes conserved across most bacteria, as well as a more variable set of genes [17]. The core genes coordinate the assembly and maintenance of the bacterial outer membrane. Many of the variable σ^E^ regulon members are critical for virulence in important pathogens [18–21].

The structure of *Ec* σ^E^ bound to the cytoplasmic portion of its anti-σ RseA revealed that, despite little primary sequence identity, domains 2 and 4 of σ^E^ (σ^E^_2_ and σ^E^_4_, respectively) share striking structural similarity to the corresponding domains of *Taq* σ^A^ (σ^A^_2_ and σ^A^_4_; [22]). Domain 4 of all primary σs, which contains a helix-turn-helix DNA binding motif, recognizes the 6–base-pair (bp) −35 consensus TTGACA [4,23], while *Ec* σ^E^_4_ is thought to directly recognize the 7-bp −35 element GGAACTT [17]. Taken together, this suggests that the different groups of σ factors share the same general mechanisms of −35 element binding, but that residue changes on the surface of the recognition helix account for differences in promoter specificity. Previous studies have revealed the molecular details of how domain 4 of the group I σ factor *Taq* σ^A^ recognizes its −35 consensus promoter element [4]. To better understand the structural basis for group IV σ factor promoter specificity, we solved the 2.3-Å resolution crystal structure of *Ec* σ^E^_4_ bound to its −35 consensus promoter element. The structure reveals that, despite the structural similarity with *Taq* σ^A^_4_, *Ec* σ^E^_4_ recognizes its −35 element in a distinct manner. Conserved sequence elements of the σ^E^ −35 element, including the most highly conserved 'AA' of the GGAACTT motif, are not involved in direct interactions between the protein and the unique edges of the DNA bases. Instead, these DNA elements induce a specific DNA geometry that is required for σ^E^_4_ binding. Sequence analysis of other group IV σs and their cognate −35 elements indicates that this principle of −35 element recognition is a conserved feature of −35 element recognition by group IV σ factors.

## Results

### Crystallization and Structure Determination

**Figure 1:**
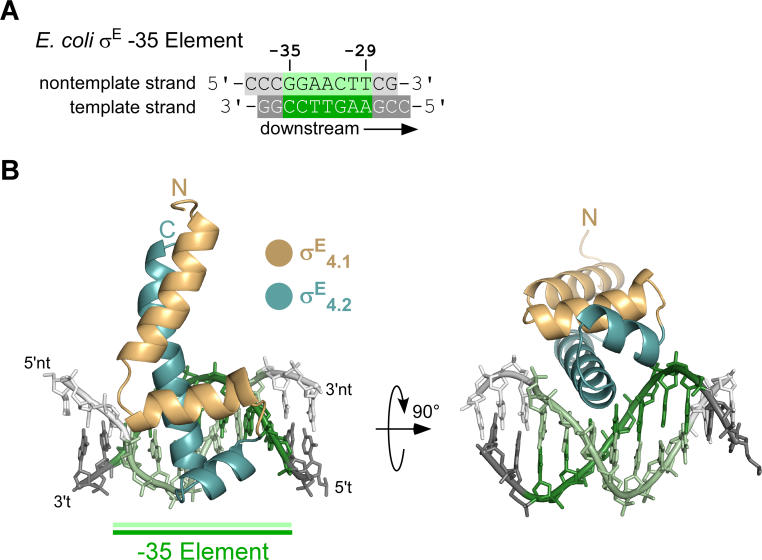
Overview of *Ec* σ^E^_4_/−35 Element DNA Structure (A) Synthetic 12-mer oligonucleotides use for crystallization. The black numbers above the sequence denote the DNA position with respect to the transcription start site at +1. The −35 element is colored light green (nontemplate strand) and dark green (template strand). The flanking bases are colored light gray (nontemplate strand) and dark gray (template strand). (B) Two views of the *Ec* σ^E^_4_/−35 element DNA complex, related by a 90° rotation about the horizontal axis as shown. The protein is shown as an α-carbon backbone ribbon, with σ^E^_4.1_ colored yellow and σ^E^_4.2_ colored light blue. The DNA is color coded as in (A).

We performed vapor diffusion crystallization trials with *Ec* σ^E^_4_ (residues 122 to 191) in complex with DNA fragments corresponding to the *Ec* σ^E^ consensus −35 promoter sequence GGAACTT [17]. Thin rectangular crystals grown using a 12-bp DNA fragment (Figure 1A) diffracted to 2.3 Å-resolution (see Materials and Methods and Table 1). The structure was determined by molecular replacement using both a model of *Ec* σ^E^_4_ from the *Ec* σ^E^/RseA complex structure [22] and the 6-bp −35 element from the *Taq* σ^A^_4_/DNA structure [4] in search models. The crystals contained two σ^E^_4_/DNA complexes per asymmetric unit, with a solvent content of 65%. Iterative model building and crystallographic refinement converged to an *R*/*R*_free_ of 0.241/0.253 (Table 2).

**Table 1:**
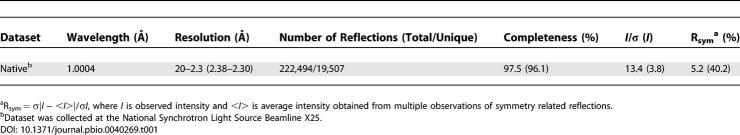
*Ec* σ^E^_4_/DNA Diffraction Data

**Table 2:**
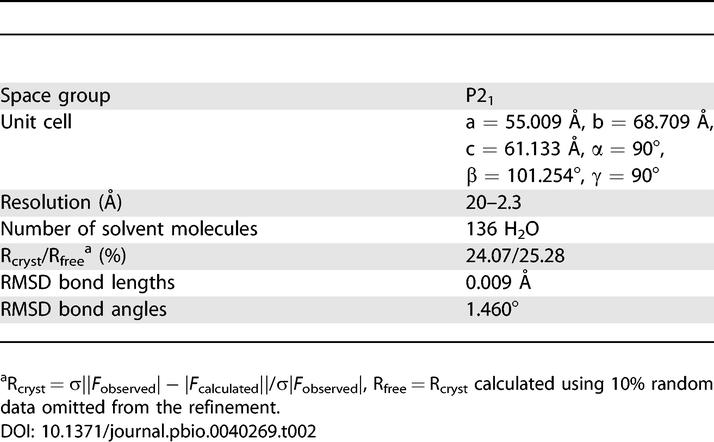
*Ec* σ^E^_4_/DNA Crystallographic Analysis and Refinement (against Native Dataset)

### Overall Structure

Two σ^E^_4_ molecules in the asymmetric unit each bound a separate DNA fragment. As anticipated, the recognition helix of the σ^E^_4_ helix-turn-helix motif bound in the major groove of the −35 element (Figure 1B). The crystallographically related DNA helices packed head-to-tail, forming a pseudo-continuous double helix with the 1 bp overhangs forming Hoogstein base pairs with the adjacent double helices.

### σ^E^_4_–DNA Interactions

**Figure 2:**
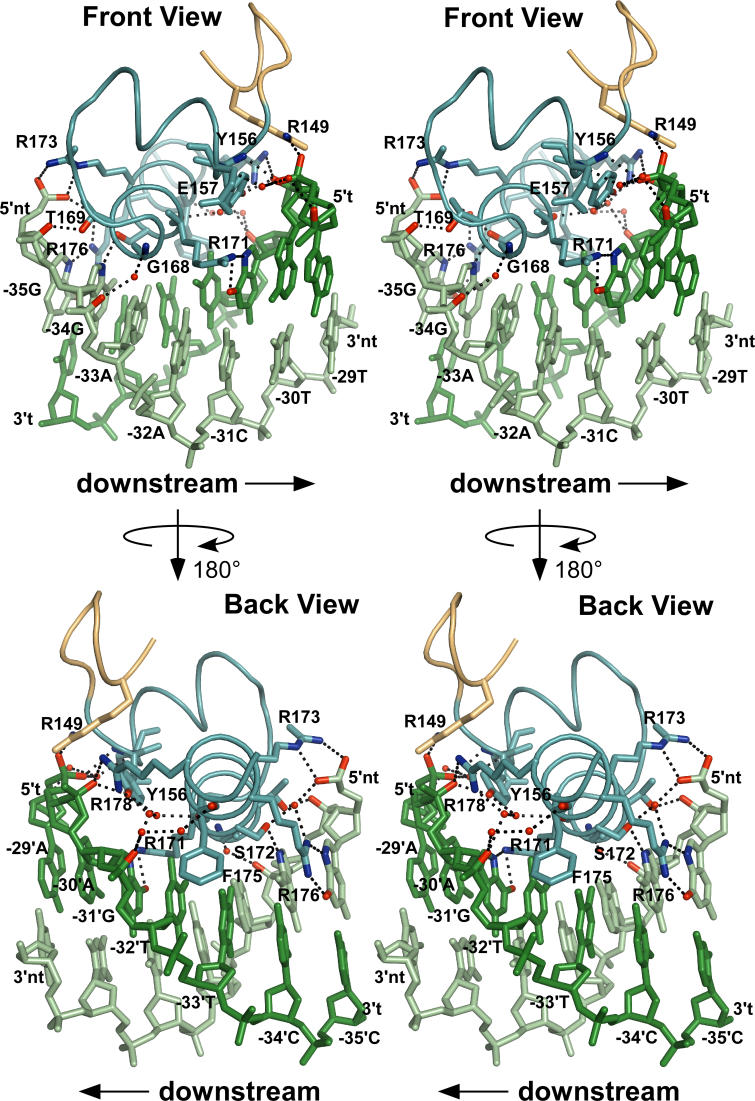
*Ec* σ^E^_4_/DNA Contacts; Structural View Two stereo views (front and back) of the *Ec* σ^E^_4_/−35 element DNA complex, related by a 180° rotation about the vertical axis as shown. The protein is shown as an α-carbon backbone worm, with σ^E^_4.1_ colored yellow and σ^E^_4.2_ colored light blue. Side chains are shown for those residues that make protein–DNA contacts. Carbon atoms of the side chains are colored as the backbone, except atoms involved in polar contacts with the DNA are colored (nitrogen atoms, blue; oxygen atoms, red). The DNA is color-coded as in Figure 1A, except atoms involved in polar contacts with the protein are colored (nitrogen atoms, blue; oxygen atoms, red). Water molecules are indicated with red spheres. Dashed black lines indicate hydrogen bonds or salt bridges.

**Figure 3:**
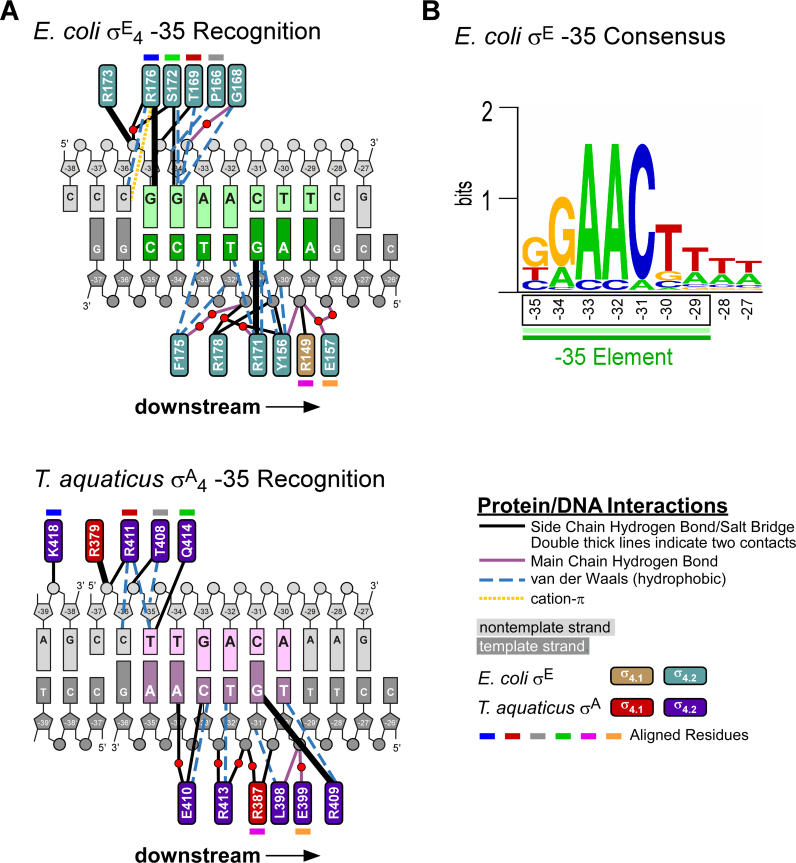
*Ec* σ^E^_4_/DNA Contacts; Schematic View (A) Schematic representation of σ_4_–DNA interactions for *Ec* σ^E^_4_ (top) and *Taq* σ^A^ (bottom; [4]). The nontemplate/template strand DNA is colored light gray/dark gray (respectively), except the −35 element is colored light green/dark green (for *Ec* σ^E^_4_) or pink/magenta (for *Taq* σ^A^). Colored boxes denote protein residues. Color-coding for the proteins, as well as the meaning of the lines indicating interactions, is explained in the legend (lower right). Double thick solid black lines indicate two hydrogen bonds with the same residue. Water molecules mediating protein–DNA contacts are shown as red circles. (B) Sequence logo denoting sequence conservation within the *Ec* σ^E^_4_ −35 element [17,51].

Protein–DNA interactions, which occur exclusively within the major groove, extend from −29 to −36, spanning the entire −35 element as well as one base of upstream DNA (Figures 2 and 3A). The protein anchors itself to the DNA by direct and water-mediated side chain and main chain interactions with the phosphate backbone on the nontemplate strand from −33 to −35 and the template strand from −29′ to −32′ [throughout this paper, DNA bases will be numbered as in Figure 3A, where negative numbers denote base pairs upstream of the transcription start site. Unprimed numbers denote the nontemplate (top) DNA strand, while primes denote the template (bottom) strand]. Specific protein–DNA base interactions occur through direct hydrogen bonds and van der Waals forces (Figures 2 and 3A). In addition, there is one cation–π interaction between R176 and −36.

Interestingly, the primary base-specific protein–DNA interactions occur at only three positions of the 7-bp −35 element (all Guanines), −35, −34, and −31′ (Figure 3A). The upstream edge of the −35 element is recognized through a series of hydrogen bonds and van der Waals interactions, mostly between R176 and S172 and the guanine bases at −35 and −34. R176 forms two hydrogen bonds with the −35G. In addition, R176 forms a cation–π interaction with the −36 DNA base, creating a stair motif along with the −35 hydrogen bonds [24,25]. S172 forms direct hydrogen bond and van der Waals interactions with the −34G. The protein–DNA base-specific interactions at the −31′ position are almost exclusively from R171, which makes two hydrogen bonds and one van der Waals interaction with the −31′G.

In contrast to the numerous base-specific interactions at the −35, −34, and −31′ positions, the −33 and −32 positions each contain only one base-specific contact, in the form of van der Waals interactions between the thymidine C5-methyl groups at −33′ and −32′ with F175 and R171, respectively (Figure 3A). The structure reveals no base-specific protein–DNA interactions at the −30 and −29 positions.

### Geometry of the σ^E^_4_ −35 Element DNA

**Figure 4:**
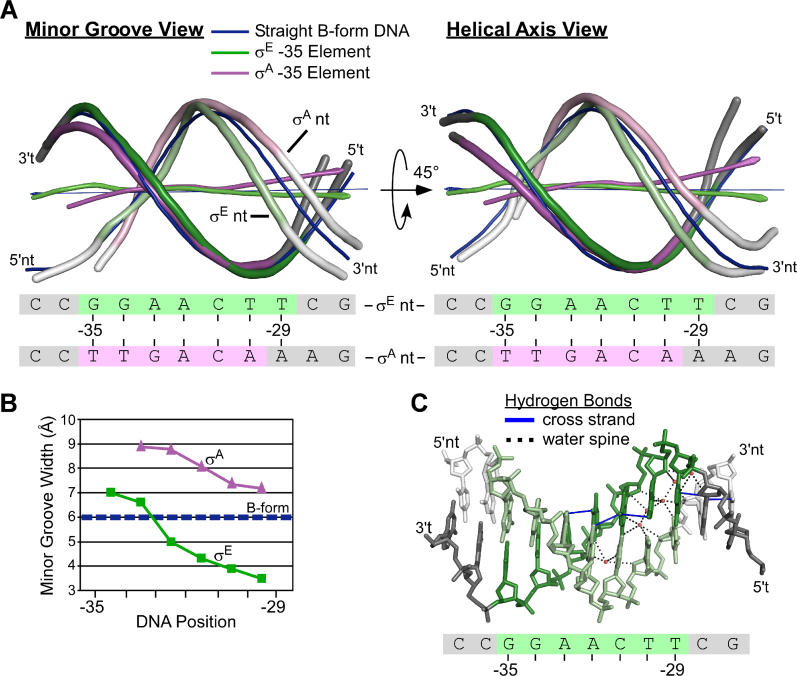
*Ec* σ^E^ −35 Element DNA Geometry (A) Cartoon views of the DNA backbone geometry. The DNA was aligned using the template strand DNA from −35′ to −30′, giving an RMSD of 0.839 over 30 atoms for *Ec* σ^E^_4_/DNA and *Taq* σ^A^_4_/DNA. Straight B-form dsDNA is blue, *Ec* σ^E^ −35 element DNA is green, while *Taq* σ^A^ −35 element DNA is magenta. The paths of the DNA helical axes, calculated using Curves (http://www.ibpc.fr/UPR9080/Curindex.html), are also shown. (B) Graph showing the DNA minor groove width (calculated using 3DNA) for B-form DNA (blue), *Ec* σ^E^_4_ −35 element DNA (green), and *Taq* σ^A^ −35 element DNA (magenta; [49]). Minor groove width was calculated as the P-P distance minus 5.8 Å to take into account the radii of the phosphate groups. (C) View of the hydrogen bonds important in stabilizing the unique geometry of the downstream σ^E^ −35 element DNA. The waters participating in the spine of hydration are indicated by red spheres. Dashed black lines indicate water-mediated minor groove hydrogen bonds. Dashed blue lines indicate cross-strand hydrogen bonds formed between adjacent bases.

Over four of the −35 element positions (−33, −32, −30, −29), there are a total of only two protein–DNA-base contacts, both weak, van der Waals contacts (Figure 3A). Nevertheless, the −33 and −32 positions are the most highly conserved positions, not only in the *Ec* σ^E^ −35 consensus but also across all group IV σ factors where the promoter specificity is known (Figure 3B; [7,17]). Furthermore, genetic screens for defective transcription resulting from single nucleotide substitutions in the −35 element of the *Ec* σ^E^ homolog from Salmonella enterica serovar Typhimurium only resulted in the selection of mutants with substitutions at positions −33 and −32 [26]. Therefore, how is it that the most highly conserved and essential positions in the σ^E^ −35 element are also the same ones that lack strong protein–DNA base interactions? The answer for this apparent paradox comes from the unique DNA geometry of the σ^E^ −35 element (Figure 4).

The unique DNA geometry induced by oligo(dA) • oligo(dT) tracts, defined by the presence of four to six consecutive A • T bp, is well established [27–31]. Depending on its sequence, oligo(dA) • oligo(dT) tract DNA is rigid and straight, with a high degree of propeller twist and a very narrow minor groove. Despite not being a true oligo(dA) • oligo(dT) tract as a result of the cytosine insertion at −31, the σ^E^ −35 element DNA is relatively straight (Figure 4A), with a high degree of propeller twist (Figure S1), and the minor groove width begins to narrow at the start of the −33/−32 AA (Figure 4B). The narrow minor groove is stabilized by a network of cross-strand hydrogen bonds between adjacent DNA bases, along with a spine of hydration consisting of water-mediated hydrogen bonds between the two strands (Figure 4C). The AA at −33/−32 is the most highly conserved feature of the σ^E^ −35 consensus. After the −31 cytosine insertion, the consensus comprises TT (−30/−29). Furthermore, there is a continued run of two additional conserved Ts at −28/−27 (Figure 3B; [17]).

Interestingly, the nucleosome structure [32] contains a stretch of DNA, GAAGTT, similar in sequence to −34 to −29 (GAACTT) of the *Ec* σ^E^ −35 element (Figure S2). Similar to *Ec* σ^E^ −35 element DNA, the nucleosome DNA cannot be classified as a typical oligo(dA) • oligo(dT) tracts as a result of the non-A/T base, yet it too displays the hallmark DNA geometry, such as a very narrow minor groove (Figure S2B). The presence of similar DNA geometry in two different structural contexts strongly suggests that the oligo(dA) • oligo(dT)–like DNA geometry found in the *Ec* σ^E^ −35 element DNA complex is an intrinsic property of the DNA sequence and not due to protein induced conformational changes.

The absence of strong, base-specific protein–DNA interactions at the −33, −32, and −30 to −27 positions (Figure 3A) is conspicuous in light of the high DNA sequence conservation, particularly at the −33/−32 positions (Figure 3B). This, combined with the observation that the DNA sequence induces a unique geometry in the −35 element DNA (Figure 4), strongly suggests that the DNA sequence is conserved at these positions to set up the global conformation of the DNA, and that this DNA conformation is essential for σ^E^_4_ binding.

In this light, the results of the previous genetic screen [26] make good sense. Individual mutations at positions other than the −33 and −32 could be compensated for by both the binding interactions at other −35 element positions and by protein–DNA backbone interactions, which would not be lost at the mutated position. However, substitutions at the −33/−32 positions, which disrupt the highly conserved AA, would in turn disrupt the global DNA geometry necessary for σ^E^_4_ binding.

### Comparison of σ^E^_4_ and σ^A^_4_ −35 Element Recognition

**Figure 5:**
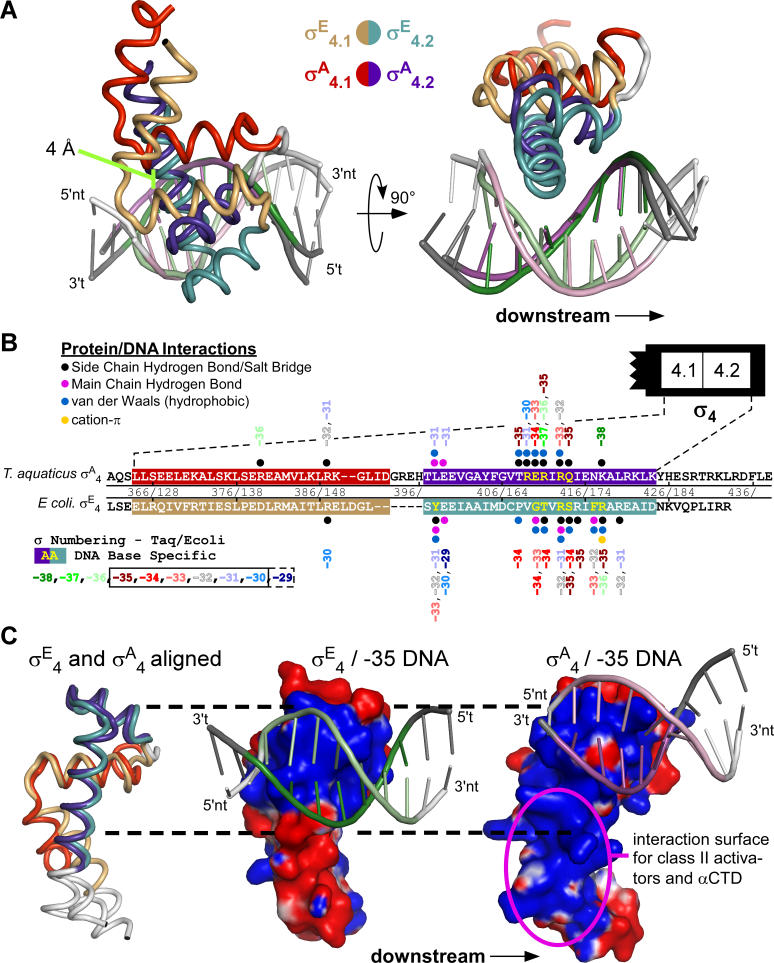
Structural Comparisons of *Ec* σ^E^_4_ and *Taq* σ^A^_4_ −35 Element Recognition (A) *Ec* σ^E^_4_/−35 element DNA and *Taq* σ^A^_4_/−35 element DNA complexes were aligned using the template strand DNA from −35′ to −30′, giving an RMSD of 0.839 over 30 atoms. The two views are related by a 90° rotation about the horizontal axis as shown. Proteins are shown as α-carbon backbone worms, color-coded as shown. The *Ec* σ^E^ −35 element DNA is colored light green (nontemplate strand) and dark green (template strand). The *Taq* σ^A^ −35 element is colored pink (nontemplate strand) and magenta (template strand). (B) Comparison of the *Ec* σ^E^_4_ and *Taq* σ^A^_4_ protein–DNA interactions. The Cα-backbone of *Ec* σ^E^_4_ and *Taq* σ^A^_4_ were aligned using *Ec* σ^E^_4_ residues 137 to 150 and 155 to 182 with *Taq* σ^A^_4_ residues 375 to 388 and 397 to 424, giving an RMSD of 1.00 Å over 42 atoms. Protein residue numbering is shown between the sequences (*Taq*/*Ec*). Residues in σ_4.1_ are highlighted in red/yellow (*Taq* σ^A^/*Ec* σ^E^) and those in σ_4.2_ are colored purple/blue. Red dots denote protein residues that make base-specific DNA contacts. Colored dots denote protein residues that make DNA contacts. Black dots denote hydrogen bonds (less than 3.2 Å) or salt bridges (less than 4.0 Å) originating from the protein side chain. Magenta dots denote hydrogen bonds originating from the protein main chain. Blue dots denote van der Waals (hydrophobic) contacts (less than 4.0 Å). Yellow dots denote cation–π interactions. The positions along the DNA that are contacted by each residue are indicated above and below the contact circles. (C) The protein α-carbon backbones of *Ec* σ^E^_4_ and *Taq* σ^A^_4_ were aligned as described in (B). The superimposed proteins, shown as α-carbon backbone worms, are shown on the left, color-coded as in (A). The *Ec* σ^E^_4_/−35 element and *Taq* σ^A^/−35 element complexes are shown separately (middle and left, respectively). In these views, the proteins are shown as molecular surfaces, color-coded according to electrostatic surface potential. The DNAs are shown as phosphate-backbone ribbons, with bases indicated schematically as sticks.

Superposition of the DNA from the *Ec* σ^E^_4_ and *Taq* σ^A^_4_ [4] −35 element complexes reveals that *Ec* σ^E^_4_ binds 4 Å further into the major groove than the group I σ factor *Taq* σ^A^_4_, allowing *Ec* σ^E^_4_ to form more extensive interactions with the DNA (Figure 5A). In addition, this shift extends the DNA recognition surface of the protein toward the C-terminus of the helix-turn-helix motif recognition helix of *Ec* σ^E^_4_ (Figure 5B). For example, even though both promoters have a G at −31′, with *Taq* σ^A^_4_ it is recognized by R409 and with *Ec* σ^E^_4_ it is recognized by R171, which is four residues (one helical turn) further toward the C-terminus in the aligned sequences.

Furthermore, the aligned residues *Taq* σ^A^_4_ K418 and *Ec* σ^E^_4_ R176 contact the DNA at different positions. Whereas *Taq* σ^A^_4_ K418 makes contacts upstream of the *Taq* σ^A^ −35 element at −38, *Ec* σ^E^_4_ R176 forms many important interactions within the σ^E^_4_ −35 element at −35. Interestingly, *Taq* σ^A^_4_ makes one van der Waals and four hydrogen bond protein–DNA contacts upstream of the −35 element at −36 and −38, whereas, *Ec* σ^E^_4_ only makes one van der Waals and one cation–π interaction with the nearby −36 DNA base. In essence the 4-Å shift causes the regions of *Taq* σ^A^_4_ that were involved in upstream non-promoter element contacts to be involved in sequence specific −35 element contacts in the *Ec* σ^E^_4_/DNA structure. For example, in both structures aligned residues K418/R176 (*Taq* σ^A^_4_/*Ec* σ^E^_4_), T408/P166, R411/T169, and Q414/S172 make up the majority of the upstream nontemplate strand interactions. However, in the case of *Ec* σ^E^_4_ they all make interactions within the −35 element at −35 and −34, whereas in *Taq* σ^A^_4_ they make interactions mostly upstream of the −35 element (−38 to −35). Similarly, the aligned residues R387/R149, L398/Y156, and E399/E157 interact in both structures with the downstream template strand DNA backbone. However, in *Ec* σ^E^_4_ R149 and E157 make their contacts 1 to 2 bp farther downstream than *Taq* σ^A^_4_ R387 and E399 (Figure 5B).

In contrast to the genetic screen for nucleotide substitutions in the σ^E^ −35 element, which only found decreased transcription from mutations at two of the seven promoter positions (−33 and −32; [26]), systematic mutational studies of the *Ec* σ^70^ −35 element have shown decreased transcription from mutations at five of the six promoter positions (−35 to −31; [33]). The two structures also show major differences in the geometry of the −35 element DNA. Whereas *Taq* σ^A^_4_ bends its −35 element, the protein-bound *Ec* σ^E^_4_ −35 element DNA is relatively straight (Figure 4A). Unlike the σ^70^ −35 element, the *Ec* σ^E^ −35 element itself adopts a unique DNA geometry (described above) that leads to a rigid, straight DNA segment. In fact, unlike the primary σs, which utilize the flexibility of its −35 element DNA, *Ec* σ^E^ appears to use the rigidity of its −35 element DNA sequence to increase specificity.

Superposition of the proteins from the *Ec* σ^E^_4_ and *Taq* σ^A^_4_ −35 element complexes highlights the significant differences in the positioning of the −35 element DNA with respect to the protein, and the different properties of the protein surfaces available for interacting with other proteins bound to the upstream DNA (Figure 5C). Conserved, basic residues of the group I σ domain 4 are key targets for interacting with acidic residues of class II transcriptional activators that bind just upstream of the −35 element [4,34,35]. The role of transcriptional activators in controlling σ^E^ transcription is largely unknown.

### Implications for −35 Element Recognition by Other Group IV σ Factors

The primary sequences of the group IV σ factors are much more divergent from each other than the members of the other σ^70^-family subgroups. Furthermore, some genomes contain over 60 group IV σ factors, each of which can recognize unique, but overlapping, sets of promoter sequences. Nevertheless, the various group IV σ factors generally share a high degree of conservation in their −35 element sequences, implying that the less conserved −10 element sequences provide the primary basis for promoter specificity between the different group IV σs, especially within the same species [7,36,37]. Therefore, the mechanism of −35 element recognition revealed in the *Ec* σ^E^_4_/DNA structure should be relevant to other group IV σ factors.

**Figure 6:**
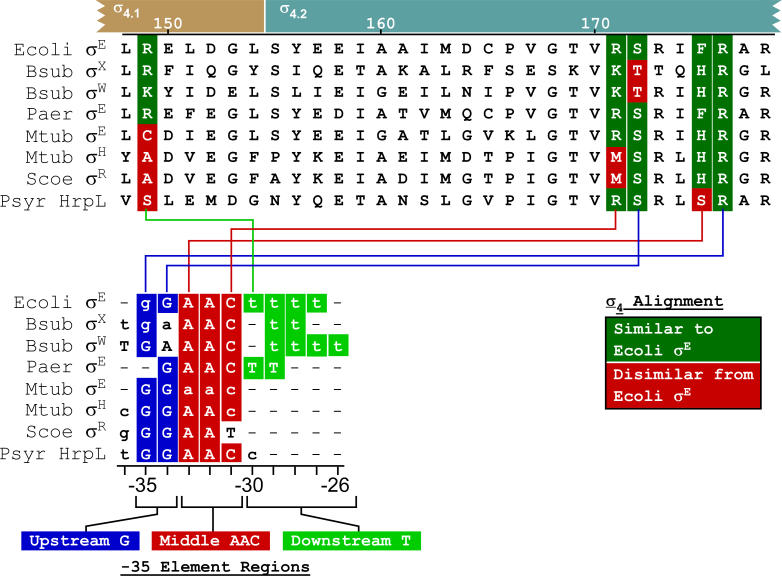
Correlation of σ_4_ and −35 Element Sequences for Several Group IV σ Factors The top shows a sequence alignment of the proposed −35 element DNA binding region of several group IV σ factors. The residue positions that are important in −35 element DNA recognition in the *Ec* σ^E^_4_/−35 element DNA structure are highlighted green (similar to *Ec* σ^E^) or red (dissimilar to *Ec* σ^E^). The bottom shows the alignment of the known −35 consensus sequences from several group IV σ factors. The three −35 element regions are highlighted with the upstream G region (blue), the middle AAC motif (red), and the downstream T rich region (green). Lines connecting the two alignments indicate protein residue–DNA base interactions important for −35 element recognition in the *Ec* σ^E^_4_/DNA structure.

Partial to fully characterized regulons have been described for at least eight group IV σs: *Ec* σ^E^ [17], *Bacillus subtilis (Bsu)* σ^X^ [38], *Bsu* σ^W^ [39], *Pseudomonas aeruginosa (Paer)* σ^E^ [37,40], *Mycobacterium tuberculosis (Mtub)* σ^E^ [41], *Mtub* σ^H^ [42], *Streptomyces coelicolor (Scoe)* σ^R^ [43], and *Pseudomonas syringae (Psyr)* HrpL [44]. When considering the −35 elements recognized by these group IV σs together, the −35 element can clearly be divided into three distinct regions. The first is an upstream G region, the second is the previously recognized AAC motif [7], and the third is a less well-conserved downstream T-tract (Figure 6 and Figure S3). The differences and similarities between the consensus −35 elements recognized by these group IV σs can be directly explained from the σ^E^_4_ sequence alignments in light of the σ^E^_4_/DNA structure (Figure 6). For example, when consensus sequences for the −35 elements are aligned by the highly conserved AAC motif, all but one of them contain a G at the position equivalent to the *Ec* −35 position. In the structure, this position is recognized by *Ec* σ^E^ R176, which is conserved across all the Group IV σs. At the −34 position of the promoter consensus, the occurrence of G or A correlates perfectly with the presence of S or T (respectively) at amino acid position 172.

In the *Ec* σ^E^_4_/−35 element structure, the face of the phenyl-ring of F175 makes van der Waals interactions with the C5-methyl group of the T opposite the absolutely conserved A at position −33. Consistent with this, all of the Group IV σs except for *Psyr* HrpL have either an F or an H (which could contribute similar van der Waals interactions) at the equivalent amino acid position.

Amino acid residue R171 of σ^E^_4_ donates a hydrogen bond to the G opposite the highly conserved C at position −31. Correlating with the conservation of C at this position of the promoter is the occurrence of amino acid residues R or K (which could also donate a hydrogen bond to the complementary G). In the two exceptions, *Mtub* σ^H^ and *Scoe* σ^R^ have M at this amino acid position, and the *Scoe* σ^R^ consensus has a T at this position, while the *Mtub* σ^H^ −35 element has a very weak C/T at this position. Even the downstream T rich sequence, whose primary residue-specific interaction is with R149, is found only in the consensus of those σ factors (*Bsu* σ^X^, *Bsu* σ^W^, *Paer* σ^E^) which contain an R or equivalent residue at this position. These correlations suggest that the mechanism of binding found in the *Ec* σ^E^_4_/DNA structure can be generalized to other group IV σ factors.

### Conclusion

Despite similar function and secondary structure, the group I and IV σ factors recognize their −35 elements using distinct mechanisms. The group IV σ factor *Ec* σ^E^_4_ binds 4 Å further into the major groove than the group I σ factor *Taq* σ^A^_4_, making more extensive contacts. Unlike *Taq* σ^A^_4_, *Ec* σ^E^_4_ does not bend the DNA. Instead, conserved sequence elements of the σ^E^ −35 promoter induce DNA geometry characteristic of oligo(dA) • oligo(dT)−tract DNA, including pronounced minor groove narrowing. For this reason, the highly conserved AA at −33/−32 is essential for −35 element recognition by σ^E^_4_, even in the absence of direct protein interactions with the DNA bases. It appears that these principles of σ^E^_4_/−35 element recognition can be applied to a wide range of other group IV σ factors.

## Materials and Methods

### Cloning, expression, and purification of *Ec* σ^E^_4_.

The gene encoding *Ec* σ^E^_4_ (residues 122 to 191) was PCR subcloned from pLC31 [22] into the NdeI/BamHI sites of the pET-15b expression vector (Novagen, Madison, Wisconsin, United States), creating pWJL3. The plasmid was transformed into *Ec* BL21(DE3)pLysS cells, and transformants were grown at 37 °C in LB medium with amplicillin (100 μg/ml) to an OD_600_ of 0.4 to 0.6. Protein expression was induced with 1 mM IPTG for 4 h. Cells containing the overexpressed protein were harvested and resuspended in lysis buffer (20 mM Tris-HCl [pH 8.0], 0.5 M NaCl, 5% glycerol, 0.1 mM EDTA, 5 mM imidazole [pH 8.0], 0.5 mM β-ME, and 1 mM phenylmethylsulfonylfluoride). Cells were lysed using a sonicator and clarified by centrifugation. Supernatants were applied to 2 × 5 ml of Ni^2+^-charged HiTrap metal-chelating columns (Amersham Biotech [GE Healthcare], Piscataway, New Jersey, United States). Lysis buffer with 20 mM imidazole was used to wash the column, followed by elution of the tagged protein using lysis buffer with 250 mM imidazole. To remove the (His)_6_-tag, samples were diluted into thrombin digestion buffer (20 mM Tris-HCl [pH 8], 0.15 M NaCl, 5% glycerol, 5 mM CaCl_2_, and 0.5 mM β-ME) and treated with thrombin (500 μ g/100 mg protein) at 4 °C. To separate the cleaved (untagged) protein from the thrombin and uncleaved, (His)_6_-tagged protein, the sample was reapplied to the Ni^2+^-charged HiTrap column in tandem with a 1 ml Benzamidine FF HiTrap column (Amersham), and the flow-through was collected. The sample was then precipitated using ammonium sulfate (60 g/100 ml sample), centrifuged, and resuspended in gel filtration buffer (20 mM Tris-HCl [pH 8], 0.5 M NaCl, 5% glycerol, and 1 mM DTT). The resuspended sample was applied to a Superdex 75 gel filtration column (Amersham) equilibrated with gel filtration buffer. The eluted *Ec* σ^E^_4_ was concentrated to 30 mg/ml by centrifugal filtration (ViaScience, Hanover, Germany) and exchanged into a low salt crystallization buffer (20 mM Tris-HCl [pH 8], 0.2 M NaCl, 5% glycerol, 0.1 mM EDTA, and 1 mM DTT). Since *Ec* σ^E^_4_ rapidly precipitated at room temperature when in a low salt buffer (less than 0.3 M NaCl), all subsequent steps were done in the cold room using prechilled supplies. The final purified protein product was aliquoted, flash frozen, and stored at −80 °C. Electrospray mass spectrophotometry was used to confirm the mass of the purified product (8,427 Da).

### Nucleic acid preparation.

For the purposes of crystallization, several different DNA constructs were designed, based on the *Ec* σ^E^_4_ −35 consensus. Construct length and flanking bases were varied in an attempt to promote crystallization through end-to-end dsDNA contacts. Lyophilized, tritylated, single-stranded oligonucleotides (Oligos Etc., Wilsonville, Oregon, United States) were detritylated and purified on an HPLC using a Varian (Palo Alto, California, United States) Microsorb 300 DNA column [45]. The purified oligonucleotides were dialyzed into 5 mM TEAB (pH 8.5) and dried on a SpeedVac (Savant). The dried oligonucleotides were resuspended in 5 mM Na cacodylate (pH 7.4), 0.5 mM EDTA, 50 mM NaCl to a concentration of 1 mM. Equimolar amounts of oligonucleotides were annealed by heating to 95 °C for 5 min and then cooling to 22 °C at a rate of 0.01 °C/s. The annealed oligonucleotides were dried in a SpeedVac and stored at −20 °C.

### Crystallization and structure determination of the *Ec* σ^E^_4_–DNA complex.

Co-crystals were obtained by vapor diffusion by mixing the duplex DNA (Figure 1A) and *Ec* σ^E^_4_ (molar ratio 1:1.5) with the final concentration of protein at 1.8 mM (15 mg/ml). The mixture was centrifuged for 30 min, then was mixed with an equal volume of well solution (0.04 M MgCl_2_, 0.05 M Na-Cacodylate [pH 6.0], and 5% v/v 2-methyl-2,4-pentanediol). Rectangular crystals (0.3 × 0.1 × 0.06 mm) grew within 5 d. Crystals were prepared for cryocrystallography by soaking in the crystallization solution supplemented with 25% 2-methyl-2,4-pentanediol, followed by flash freezing in liquid nitrogen. A native dataset was collected to 2.3 Å at The National Synchrotron Light Source (NSLS, Brookhaven National Laboratory, Upton, New York, United States), Beamline X25 (Table 1).

The structure was solved by molecular replacement with Molrep 8.1 [46] using *Ec* σ^E^_4_ from the *Ec* σ^E^–RseA complex structure [22]. Initially, Molrep was used to search for solutions with 2 or 3 molecules per asymmetric unit. Both searches yielded a solution with two molecules of *Ec* σ^E^_4_ arranged in a symmetrical dimer (Molrep Corr = 0.252). Though there were some slight clashes between the flexible N- and C-term regions, the crystal symmetry related molecules did not clash and in fact stacked upon one another in one direction. Additionally, there was room for the dsDNA. However, when this solution was used to generate an electron density map there was no observable density for the DNA. In an effort to improve the solution, the two-molecule dimer was used as a search model to generate a new Molrep solution (Molrep Corr = 0.439), which yielded some clear dsDNA density. Molrep was further used to improve the dsDNA density by keeping the *Ec* σ^E^_4_ dimer fixed and doing two tandem molecular replacement searches using the 6-bp −35 element from the Taq σ^A^_4_/DNA structure ([4]; first DNA: Molrep Corr = 0.464 and second DNA: Molrep Corr = 0.475). In addition to placing the dsDNA into the previously seen DNA density, it extended the density one or two bases past the DNA search model. The solution was further improved by using a 1-bp register offset between the two search model DNAs, to generate a 7-bp DNA which was used to do two tandem Molrep molecular replacement searches (first DNA: Molrep Corr = 0.469 and second DNA: Molrep Corr = 0.487). CNS v1.1 [47] was then used to perform density modification, giving an improved electron density map in which clear density could be seen for the entirety of both dsDNAs, excluding the overhanging base at the downstream end of the DNA. The final DNA was built using a starting template of straight B-form dsDNA corresponding to the crystallization oligos (constructed using Namot2; http://namot.sourceforge.net). Model building was done using O v9.0.7 [48] and refinement using CNS v1.1 (Table 2).

Protein–DNA contacts were analyzed using the program CONTACT, followed by geometric verification using PyMOL v0.98 (http://www.pymol.org). Cation−π interactions were visualized using a custom PyMOL script based on previously determined geometric criteria [25]. DNA geometry was analyzed using 3DNA v1.5 [49] and Curves v5.1 (http://www.ibpc.fr/UPR9080/Curindex.html). Electrostatic surfaces were calculated using APBS: Adaptive Poisson-Boltzmann Solver [50]. All structural figures were prepared using PyMOL.

## Supporting Information

Figure S1 Comparisons of *Ec* σ^E^_4_ and *Taq* σ^A^_4_ −35 Element DNA Geometry (A) Propeller twist, (B) DNA buckle, (C) curvature, and (D) major groove width calculated using 3DNA.
(569 KB TIF)

Figure S2 Comparison of *Ec* σ^E^_4_ −35 Element DNA and Nucleosome DNA (A) The nucleosome structure contains a sequence similar to the *Ec* σ^E^_4_ −35 Element DNA. Both DNA sequences contain an AA-tract followed by a non-A/T base and then a TT-tract. Despite the non-A/T base, both structures contain narrow minor grooves, which are characteristic of oligo(dA) • oligo(dT) tracts. The DNA structures were aligned using the template strand phosphates. The minor groove narrowing is evident from the location of the non-template strand DNA relative to B-form DNA. The *Ec* σ^E^_4_ −35 element DNA is in green and the nucleosome DNA orange.
(B) Graph showing the DNA minor groove width (calculated using 3DNA) for B-form DNA (blue), *Ec* σ^E^_4_ −35 element DNA (green), and nucleosome DNA (orange). Minor groove width was calculated as the P-P distance minus 5.8 Å to take into account the radii of the phosphate groups.
(2.7 MB TIF)

Figure S3 Correlation of σ_4_ and −35 Element Sequences, along with the −10 Element Consensus, for Several Group IV σ Factors The top shows a sequence alignment of the proposed −35 element DNA binding region of several group IV σ factors. The residue positions that are important in −35 element DNA recognition in the *Ec* σ^E^_4_/−35 element DNA structure are highlighted green (similar to *Ec* σ^E^) or red (dissimilar to *Ec* σ^E^). The bottom shows the alignment of the known −10 (right) and −35 (left) consensus sequence logos from several group IV σ factors. The three −35 element regions are highlighted with the upstream G region (blue), the middle AAC motif (red), and the downstream T rich region (green). Lines connecting the two alignments indicate protein residue–DNA base interactions important for −35 element recognition in the *Ec* σ^E^_4_–DNA structure. Despite being more divergent then the −35 elements it is still possible to generate a proposed −10 element alignment. Possible regions of similarity within the −10 elements have been highlighted in light blue, magenta, and gray. The single base change thought responsible for the differential gene regulation between *Bsu* σ^X^ and *Bsu* σ^W^ is indicated with a red arrow. The column to the right of the sequence logos contains the signal and mechanism of regulation for each σ factor.
(1.7 MB TIF)

### Accession Numbers

Structure coordinates and structure factors from the *Ec* σ^E^_4_/DNA crystals have been deposited in the Protein Data Bank (http://www.rcsb.org/pdb) under ID code 2H27. The Protein Data Bank accession number for the nucleosome structure in Figure S2A is 1KX4.

## Abbreviations

bp: base pair
Bsu: Bacillus subtilis
Ec: Escherichia coli
ECF: extracytoplasmic function
Mtub: Mycobacterium tuberculosis
Paer: Pseudomonas aeruginosa
Psyr: Pseudomonas syringae
RNAP: RNA polymerase
Scoe,: Streptomyces coelicolor;
Taq: Thermus aquaticus
